# A Case of Hyponatremic Hypertensive Syndrome With Neurologic Sequelae Secondary to Unilateral Renal Artery Stenosis

**DOI:** 10.7759/cureus.68544

**Published:** 2024-09-03

**Authors:** Carson Clark, Kai Yoshinaga, Artem Tkachenko, Thomas Murphy

**Affiliations:** 1 Internal Medicine, Wright State University, Dayton, USA; 2 Internal Medicine, Dayton Veteran's Affairs Medical Center (Wright State University Internal Medicine Associate Faculty), Dayton, USA

**Keywords:** adh, hypertensive emergency, renin angiotensin aldosterone system, renal artery stenosis, hyponatremia

## Abstract

In cases of unilateral renal artery stenosis, acute exacerbations may present as hyponatremic hypertensive syndrome (HHS), a rare and highly morbid complication. Its insidious onset, low incidence, and often counter-intuitive laboratory profile can delay diagnosis and worsen outcomes. Furthermore, complications including end-organ ischemia, polyelectrolyte derangement, and hypertensive crises can occur. Herein, we present a 62-year-old man with known right renal artery stenosis who presented with HHS in hypertensive emergency with encephalopathy. Consideration of the underlying pathomechanism and careful fluid and electrolyte repletion can minimize complications and improve clinical outcomes in this highly morbid and precarious clinical syndrome.

## Introduction

Both primary and secondary hyperaldosteronism classically present with chronic hypertension, hyponatremia, and hypokalemia. However, in rare cases of secondary hyperaldosteronism from unilateral renal artery stenosis, a neurohormonal cascade involving hypertension and hyponatremia may occur [1]. This sequence was first described by Brown et al. in 1965 [1] and is termed hyponatremic hypertensive syndrome (HHS) [2]. Since then, rare case reports and one small (n=27) retrospective study have incompletely characterized the epidemiology and clinical course of the condition [3].

The syndrome can present with a variety of symptoms including polydipsia, polyuria, confusion, and seizure, as well as polyelectrolyte disturbances including hyponatremia, hypokalemia, and hypochloremia [4]. Its insidious nature, rare incidence, and often counter-intuitive laboratory profile (with elevated renin but simultaneously low sodium and potassium levels) can delay diagnosis and worsen outcomes. If untreated, hypertensive encephalopathy, arrhythmias, cardiomyopathy, and nephropathy may develop [3]. Herein, we present a 62-year-old man with HHS secondary to known unilateral renal artery stenosis, presenting with hypertensive emergency and altered mental status.

This case was presented as a poster at the 2024 Wright State University Boonshoft School of Medicine Student Research Symposium on April 4, 2024.

## Case presentation

The patient is a 62-year-old male with a past medical history of hypertension, seizure disorder, traumatic brain injury, polysubstance use disorder (benzodiazepines, tobacco, alcohol), and unilateral right-sided renal artery stenosis causing an atrophic right kidney who presented to the emergency department in hypertensive emergency following an unwitnessed loss of consciousness. The patient has poor mentation at baseline and cannot endorse any prodromal symptoms or sensations surrounding the loss of consciousness, its duration, his medication adherence, or his recent substance use. Collateral from the family reveals he was in an argument with his brother earlier in the evening. He has a distant history of seizures but none in the last 15 years and he is not currently taking any anticonvulsants. 

**Figure 1:**
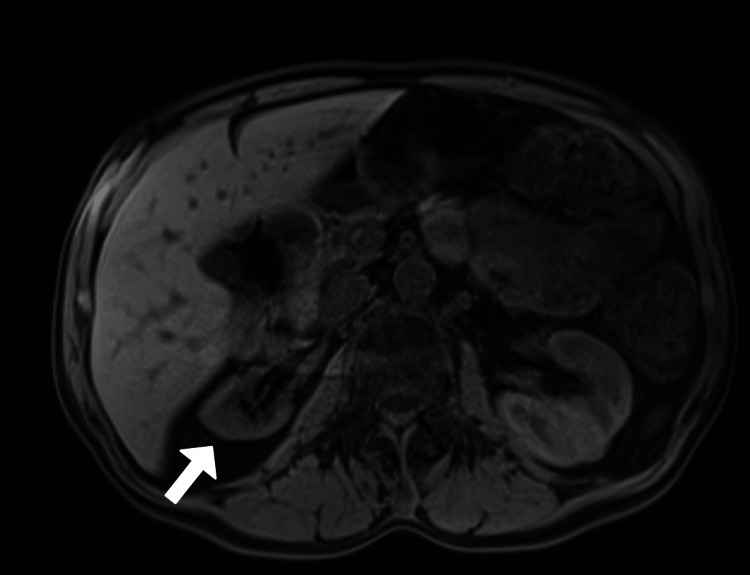
Axial T1 non-contrast MRI of the abdomen, demonstrating the patient's atrophic right kidney

Examination in the emergency department revealed hypertension to 224/146 mmHg. He was notably encephalopathic from baseline, oriented only to person, but did not exhibit any focal neurologic deficits. Laboratory workup revealed serum potassium 1.9 mmol/L (3.4-5.3mmol/L), sodium 122 mmol/L (135-148mmol/L), and chloride 83 mmol/L (96-110mmol/L). Initial troponin was 87 ng/dL (<22ng/dL) and five hours later, it was 133 ng/dL (<22ng/dL), leukocytes were 19,200 (3,500-10,900), aspartate aminotransferase was 121 U/L (0-46U/L), alanine aminotransaminase was 60 U/L (0-55U/L), blood urea nitrogen was 17 mg/dL (3-29mg/dL) and serum creatinine was 1.0 mg/dL (0.5-1.4mg/dL). Urinalysis showed large urine blood, two red blood cells/high-powered field, and four hyaline casts/high-powered field. Urine drug screen results were positive for benzodiazepines and cannabinoids. An electrocardiogram (ECG) showed no evidence of ischemic changes but prolonged QTc at 563ms. Imaging including chest X-ray (CXR) and computed tomography (CT) head did not show any new abnormalities and CT abdomen was unremarkable besides known and unchanged atrophy of the right kidney. His atrophic kidney had been followed by the nephrology service in the hospital. Magnetic resonance imaging (MRI) three months prior to presentation characterized similar levels of atrophy (Figure 1).

His acute management included initiation of a nicardipine infusion, which improved pressures from 224/146mmHg to 160/96mmHg over the course of about 24 hours, after which the nicardipine drip was titrated off. We also began fluid and electrolyte resuscitation with Lactated Ringer's solution and potassium supplementation, as well as resumed his home spironolactone 100mg/day and aliskiren 300mg/day (the patient has an allergy to ACE inhibitors) to address his polyelectrolyte derangement. A neurologic workup including MRI and electroencephalogram for seizure evaluation was unrevealing, and a cardiac workup including transthoracic echocardiogram showed no evidence of dysfunction. Troponins were presumed to be elevated secondarily to hypertensive emergency and associated left heart strain; no ischemic findings were present on ECG. The patient’s mental status improved to his baseline by day three of admission. The patient was discharged in stable condition and referred for outpatient nephrectomy of the atrophic kidney.

## Discussion

This patient’s hypertensive emergency and profound electrolyte disturbances are secondary to HHS in the setting of medication non-adherence. The pathogenesis of HHS begins with unmitigated renin release from a chronically ischemic kidney, most commonly in the setting of unilateral renal artery stenosis [2]. If the contralateral kidney is healthy, the resultant surge of the renin-angiotensin-aldosterone system can inappropriately increase blood pressures to hypertensive emergency ranges, and this, coupled with the hyper-filtrating effects of angiotensin II, causes a pressure-related natriuretic effect on the non-stenotic kidney. The natriuresis causes sodium wasting and acute volume depletion, stimulating anti-diuretic hormone (ADH) secretion. This is consistent with research demonstrating that hypothalamic baroreceptors rely more on volume status than serum osmolality for stimulation of ADH secretion [5]. It has also been postulated that heavy smoking, specifically exposure to nicotine, contributes to ADH release, though a firm mechanistic link remains to be established [6]. Regardless, the resultant surge of ADH and central thirst drive lead to retention of free water and hyponatremia. The hyperaldosteronism contributes to the expected hypokalemia. The hypertensive crisis can have its own end-organ complications such as ischemic hepatopathy, encephalopathy, or flash pulmonary edema [7].

In most cases, mainstay therapy for acute renal artery stenosis-associated HHS centers around restoration of intravascular volume [2], prevention of end-organ hypertensive crisis, and prevention of electrolyte-driven arrhythmia or seizure. Volume status should be carefully considered on a case-by-case basis, as HHS can present with flash pulmonary edema and warrant diuretic therapy instead. Volume resuscitation should take priority over blood pressure control in order to minimize the risk of acute ischemic insult to the cerebrovascular, cardiovascular, or hepatic systems [2]. Permissive hypertension is advisable during the initial period of volume repletion, as the priority in therapy is volume resuscitation [2]. After fluid resuscitation, clinicians should target a gradual reduction of blood pressure, typically achieved by continuous infusion of a calcium channel blocker. An angiotensin-converting enzyme inhibitor may be added to the patient’s home regimen to prevent HHS episodes. Long term, patients should follow closely with a nephrologist, who may recommend stenting of the renal artery, or radical unilateral nephrectomy in cases refractory to medical management and percutaneous angioplasty [8].

## Conclusions

While previous case reports and small studies have commented on the variety of complications of HHS, the incidence of neurologic sequelae such as seizures or syncopal episodes has not been clearly defined. Further research aimed at elucidating the mechanism of neurologically presenting HHS could advance diagnostic and therapeutic approaches to this disease and improve important metrics like time to fluid and electrolyte stabilization. 
